# Fetal Hemodynamic Parameters in Low Risk Pregnancies: Doppler Velocimetry of Uterine, Umbilical, and Middle Cerebral Artery

**DOI:** 10.1155/2016/1693704

**Published:** 2016-11-13

**Authors:** C. O. Figueira, F. G. Surita, M. S. Dertkigil, S. L. Pereira, J. R. Bennini, S. S. Morais, J. Mayrink, J. G. Cecatti

**Affiliations:** Department of Obstetrics and Gynecology, University of Campinas (UNICAMP), Alexander Fleming Street 101, Campinas, SP, Brazil

## Abstract

*Objective*. To elaborate curves of longitudinal reference intervals of pulsatility index (PI) and systolic velocity (SV) for uterine (UtA), umbilical (UA), and middle cerebral arteries (MCA), in low risk pregnancies.* Methods*. Doppler velocimetric measurements of PI and SV from 63 low risk pregnant women between 16 and 41 weeks of gestational age. Means (±SD) for intervals of gestational age and percentiles 5, 50, and 95 were calculated for each parameter. The Intraclass Correlation Coefficients (ICC) were also estimated for assessing intra- and intervariability of measurements.* Results*. Mean PI of UtA showed decreasing values during pregnancy, but no regular pattern was identified for mean SV. For UA, PI decreased and SV increased along gestation. MCA presented PI increasing values until 32–35 weeks. SV showed higher levels with increasing gestation. High ICC values indicated good reproducibility.* Conclusions*. Reference intervals for the assessment of SV and PI of UtA, UA, and MCA were established. These reference intervals showed how a normal pregnancy is expected to progress regarding these Doppler velocimetric parameters and are useful to follow high risk pregnancies. The comparison between results using different curves may provide insights about the best patterns to be used.

## 1. Introduction

Hemodynamic fetal study has been applied in routine ultrasonography practice since 1977, with Doppler velocimetry of the umbilical artery (UA) and its capability of diagnosing conditions of abnormal blood flow of the fetus [1]. Since then, the evaluation of intrauterine organs' perfusion and its correlation with fetal status [2] became possible, which is a very helpful method for fetal surveillance.

Technological advances in the equipment allowed the study of other fetal vessels such as middle cerebral artery (MCA) and renal arteries, improving detection of disturbances in fetus wellbeing. By monitoring the Doppler changes, it is possible to track the fetal-placental cell changes, which define the clinical picture of etiology for several fetal comorbidities. Abnormal placental perfusion in the maternal compartment results in increased blood flow resistance in the uterine artery flow velocity in waveform. Abnormal perfusion of fetal villous vascular tree is associated with decreased umbilical artery end-diastolic velocity proportional to the degree of flow impairment. Abnormal oxygen diffusion across the villous membrane is associated with a decrease in middle cerebral artery blood flow resistance [3–10].

Uterine arteries (UtA) assessment has been increasingly used as a screening method to predict the woman's risk of developing gestational diseases as preeclampsia [1, 11]. It is part of a multimarker algorithm for prediction of preeclampsia and determining potential intervention targets [11]. Elevated PI level, associated or not with early diastolic notch in the waveform, is associated with impaired uteroplacental perfusion and a higher risk of pregnancy complications as preeclampsia, fetal growth restriction (FGR), placental abruption, and adverse perinatal outcome [3, 5, 12].

Hemodynamic study is relatively recent, but many benefits of Doppler velocimetry have been demonstrated for fetal surveillance: accurate identification of risk for adverse outcome, prevention of unanticipated stillbirth, and appropriate timing of delivery [3].

Many studies assessing maternal and fetal blood flow emerged to determine values considered adequate and reference ranges have been elaborated for Doppler velocimetric parameters, mostly based on cross-sectional studies. In Brazil, the most used Doppler reference intervals are those from Arduini and Rizzo [4].

This was a study performed with low risk pregnancies to build longitudinal reference intervals for the UtA, UA, and MCA using their respective 5th, 50th, and 95th percentiles.

## 2. Methods

This was a longitudinal study with a cohort of low risk pregnant women evaluated for PI and SV (systolic velocity) of umbilical, uterine, and middle cerebral arteries' waveforms. The pregnant women were selected from March 2008 until July 2009 at the University of Campinas Medical School after answering a questionnaire to identify inclusion and exclusion criteria. A first trimester ultrasound scan confirmed the gestational age previously calculated by the last menstrual period. A scan for morphological abnormalities was also performed. Doppler velocimetry assessment of PI and SV of umbilical, uterine, and middle cerebral arteries' waveforms was performed from 16 to 36 weeks, every 4 weeks, and after 36 weeks and every 2 weeks until birth. The women developing any medical complication, who were smokers, with twin pregnancy, with fetuses diagnosed with a malformation or inadequate growth for gestational age were excluded from this study [13].

Before starting data collection, estimation of sample size was performed. Using as reference data of PI values for renal artery of the fetus in low risk pregnant women [14] and a type I error of 0.05, it was estimated that 62 cases would be necessary for generating percentiles in each gestational age.

Doppler parameters were measured using a 3.5 to 6 MHz convex transducer (*Voluson Expert 750*, GE Medical Systems or* Xario*, Toshiba) on women in the semirecumbent position in a partially darkened room. All examinations were carried out twice, either by the same ultrasonographer or by two different ones to enable the assessment of intra- and interobserver variability. Color Doppler imaging was used to identify the uterine arteries at the cervix-corporal transition of uterus and the measurements were taken at this level with a 1-2 mm sample volume. The insonation angle was as close to 0° as possible and when below 30° the measure was adjusted according to international rules [2, 13].

Umbilical artery was investigated with color Doppler ultrasonography and the waveforms were studied at the free loop portion of the umbilical cord. Middle cerebral artery was recognized by color flow mapping at a transverse section of fetal head at the level of the lesser wing of the sphenoid bone. The recordings were made at the proximal portion of the vessel [1, 2, 13]. Similarly, for uterine arteries, the insonation angle was as close to 0° as possible and when below 30° the measurement was adjusted according to international rules [2, 13].

All the records were obtained in the absence of fetal breathing and movements, with the fetal heart rate between 120 and 160 bpm. The Doppler parameters were automatically calculated by at least three consecutive waves. The high pass filter was set to 50–70 Hz.

For statistical analysis, the reference intervals and percentiles 5, 50, and 95 were established by the mean of the measurements for the quantitative parameters in each gestational age group [15]. However, the trends for increasing or decreasing values of these parameters during pregnancy were not formally performed because the main purpose was to compare reference percentiles for each gestational age with the case under investigation. The Intraclass Correlation Coefficient was also calculated for each vessel to evaluate the intra- (same ultrasonographer) and interobserver (two different ultrasonographers) variability. The intraobserver variability was measured in 268 occasions by the same examiner while the interobserver variation was taken by two different examiners at the same day and performed 129 times. Intra- and interobserver variability of these measurements were evaluated using Intraclass Correlation Coefficient. A 5% significance level was established and statistical procedures were performed using Excel® and SAS®.

The study was previously approved by the Institutional Review Board (311/2005) and all women that agreed to participate signed a written informed consent before enrollment.

## 3. Results

Sixty-three of 66 women recruited for the study from March 2008 to July 2009 met all the inclusion criteria and completed the follow-up. Three of them were excluded: diagnosis of fetal cardiac malformation, pregnant woman with Guillain Barré syndrome, and loss to follow-up. Fifty-nine women delivered in the study institution and therefore have the complete data for delivery and perinatal outcomes. Mean age of the women was 27 years; the majority was Caucasian (87%) and 47% of them were at their first pregnancy. Overweight or obese women corresponded to 45% of the sample population studied. Mean gestational age at birth was 39 weeks and mean birth weight was 3175 g. Preterm birth rate was 8.5% and all the newborns had Apgar score above 7 at the fifth minute. Complete clinical characteristics, pregnancy, and neonate outcomes are shown in Table 1.

Table 2 shows mean values and standard deviation (SD) of SV and PI of UtA, UA, and MCA according to gestational age intervals. Values of SV of UA increased during pregnancy and its PI decreased. For the MCA, the SV also increased as were PI values until 32–35 weeks, decreasing afterwards.

**Figure 1:**
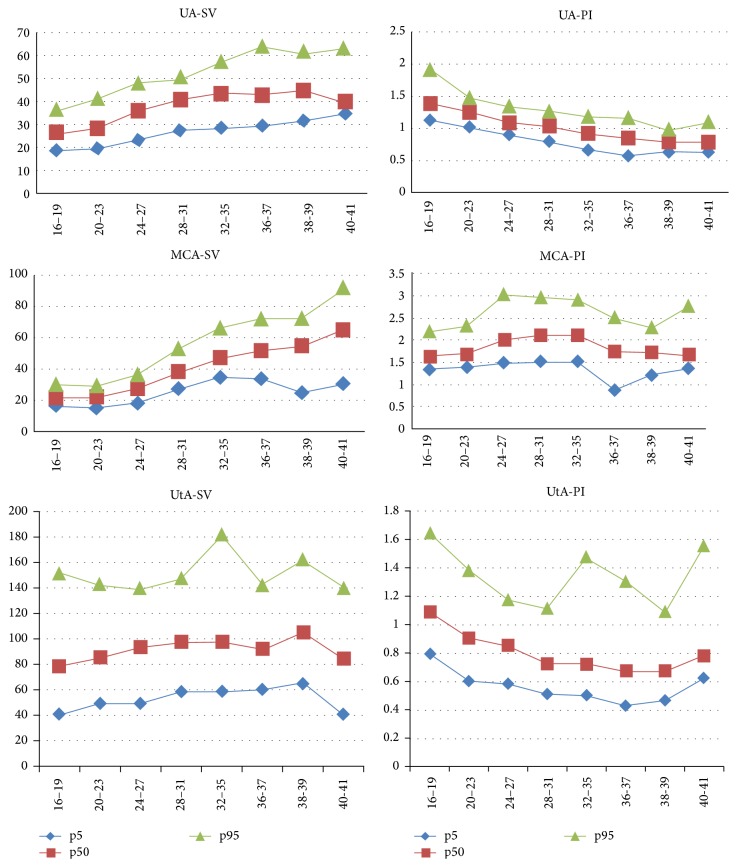
Longitudinal distribution of SV and PI of UA, MCA, and UtA.

Table 3 describes the 5th, 50th, and 95th percentiles of SV and PI of the three vessels studied and the graphic presentations of these parameters are in the correspondent Figure 1.  Table 4 shows the intra- and interobserver variability of the Doppler velocimetric parameters evaluated with their Intraclass Correlation Coefficients (ICC). Except for the values of MCA for PI and of UtA for SV in the interobserver variability, all the other assessments showed relatively high ICC.

## 4. Discussion

The standard normal reference ranges presented in this study have been based on the longitudinal follow-up of low risk pregnant women and have an evolutive pattern similar to those well established, with some variation in absolute values.

Several studies have already reported reference intervals for many Doppler velocimetric parameters [1, 2, 5–7, 16–21], especially the PI (that better describes the shape velocity waveforms) for UA, MCA, and UtA and SV for MCA, which are the indices mostly used for high risk pregnancies. Blood flow redistribution occurs in response to fetal distress and it is demonstrated by increased PI values of UA [14, 17, 18] and altered brain perfusion showed by lower PI values at MCA that marks the fetal response to placental insufficiency [3, 19]. Fetal anemia, on the other hand, is better diagnosed and followed through MCA's SV [7, 18, 20]. These fetal-maternal hemodynamic changes have a progress pattern and, for that reason, may be better accompanied by longitudinal reference intervals, which reflect changing patterns over time, rather than transverse reference intervals [5, 12]. The current study created reference intervals based on longitudinal follow-up.

Our results showed a decline pattern for the UA's PI, similar to other studies [10, 21, 22], however with differences between the absolute values. Some authors found higher absolute values [2, 23] that would be explained by different populations of study.

MCA's PI values increased until 28–31 weeks, with declining values afterwards. This pattern is in agreement with the results of Konje et al., Tarzamni et al., Tavares et al., and Ebbing et al. [2, 6, 10, 22]. However, all the studies, except the Norwegian one, showed lower absolute values [22]. Ertan et al. also showed lower levels, but the study started measurements at the 28th week, becoming impossible to compare the pattern of the complete range with our results [23]. As a common feature, SV increased during pregnancy for almost all the authors reporting it [6, 10, 22].

The UtA reflects the uteroplacental circulation and it is part of a prediction model for preeclampsia [24, 25]. Pulsatility index is the more often and consistently used parameter and its elevated value is an indicator of uteroplacental insufficiency [25–29]. Our study showed a trend to decreasing values for 5 and 50 percentiles, but no regular pattern for the 95th. These decreasing values are in agreement with other studies [10, 30, 31]. A point to be considered is the influence of the patient selection criteria on the PI absolute values among all the studies, considering that some of them included smokers, excluded women with a diastolic notch, or selected women only with certain placenta location. For the SV, the values increased through pregnancy and our values were considerably higher than those reported by Bahlmann et al. [31].

For the analysis of the method used, we showed adequate intraobserver ICC values for PI and SV for all the three vessels studied. The interobserver variability also indicates adequate ICC values, except for SV of uterine arteries and for PI of MCA. The latter could possibly be explained by the transducer pressure applied to the fetal head during the measurements. This study concludes that Doppler velocimetry of UtA, UA, and MCA is a method with adequate reproducibility, though it is crucial to have an accurate training to use the technique for proper results.

A positive point of this study is to have been based on a Brazilian population. Our patterns were similar to other reference intervals already reported, with small variations in the absolute values. Characteristics of the population analyzed would be responsible for the variability in these values. Despite the fact of being minor, these variations could imply some mistakes about the fetal wellbeing, although the influence of population or ethnic characteristics in pregnancy Doppler parameters should be checked [6]. Furthermore, reference ranges created with longitudinal studies provide a more accurate interpretation of Doppler parameters during pregnancy.

## 5. Conclusion

We have established a curve of reference intervals for pulsatility index and systolic velocity of the main arteries evaluated during pregnancy. The Doppler velocimetric measurements showed good reproducibility of the method. Even if these findings are considered not new, these values could perhaps be joined to other already available measurements, in order to provide more powerful and consistent reference intervals for these parameters through a meta-analysis that could be possibly performed in the near future.

## Figures and Tables

**Table 1:** Sociodemographic characteristics, parity, and neonatal outcomes of women included in the study.

Characteristics	*n*	%
*Age (years)*		
≤19	4	6.3
20–29	41	65.1
≥30	18	28.6
*Parity*		
0	30	47.6
≥1	33	52.4
*Ethnicity*		
Caucasian	55	87.3
Others	8	12.7
*BMI* ^*∗*^		
<18.5 (low)	0	0
18.5–24.9 (normal)	33	55.0
25–29.9 (overweight)	19	31.7
≥30.0 (obesity)	8	13.3
*GA at birth*		
<35	1	1.7
35-36	4	6.8
37–40	43	72.9
>40	11	18.6
*Birth weight* ^*∗∗*^		
<2500 g	2	3.5
2500–3999 g	53	93.0
≥4000 g	2	3.5
*Apgar score < 7*		
1st minute	1	1.7
5th minute	0	0

^*∗*^3 women without BMI data.

^*∗∗*^2 newborns without birthweight data.

**Table 2:** Means and standard deviation (SD) of PI and SV of uterine, umbilical, and middle cerebral artery in cohort of a low risk pregnant women.

GA (weeks)	*N*	Uterine artery		Umbilical artery		Middle cerebral artery	
		PI (±SD)	SV cm/s (±SD)	PI (±SD)	SV cm/s (±SD)	PI (±SD)	SV cm/s (±SD)
16–19	63	1.16 (±0.37)	244.91 (±949.99)	1.43 (±0.24)	26.61 (±5.60)	1.70 (±0.28)	21.78 (±4.40)
20–23	63	0.93 (±0.24)	89.69 (±32.44)	1.24 (±0.14)	29.52 (±7.08)	1.81 (±0.36)	22.93 (±5.05)
24–27	63	0.85 (±0.20)	94.28 (±31.74)	1.08 (±0.17)	35.64 (±8.73)	2.14 (±0.71)	28.36 (±6.36)
28–31	63	0.77 (±0.20)	97.36 (±26.05)	1.03 (±0.15)	39.36 (±8.03)	2.14 (±0.41)	38.42 (±7.99)
32–35	63	0.80 (±0.35)	149.93 (±277.64)	0.93 (±0.24)	44.20 (±9.65)	2.18 (±0.44)	49.22 (±9.81)
36-37	53	0.73 (±0.23)	96.13 (±26.79)	0.85 (±0.20)	44.44 (±11.10)	1.79 (±0.45)	53.24 (±13.56)
38-39	29	0.71 (±0.20)	104.41 (±28.35)	0.79 (±0.12)	45.40 (±9.26)	1.72 (±0.32)	52.98 (±13.54)
40-41	14	0.90 (±0.30)	84.84 (±30.23)	0.81 (±0.15)	44.85 (±10.87)	1.74 (±0.39)	61.25 (±19.19)

GA, gestational age; SV, systolic velocity; PI, pulsatility index; SD, standard deviation.

**Table 3:** Percentiles 5, 50, and 95 of SV and PI of uterine, umbilical, and middle cerebral arteries according to gestational age intervals.

GA (weeks)	*N*	Uterine arteries			Umbilical artery			Middle cerebral artery		
		P5	P50	P95	P5	P50	P95	P5	P50	P95
Systolic velocity (cm/s)										
16–19	63	40.25	78.08	151.25	18.36	26.14	36.16	16.24	21.71	30.36
20–23	63	49.25	85.49	142.48	19.11	28.63	41.30	15.19	22.25	29.40
24–27	63	48.98	93.33	139.17	23.30	35.88	48.37	18.53	27.98	36.61
28–31	63	58.64	97.17	147.36	27.27	40.77	49.78	27.01	38.48	53.60
32–35	63	58.40	97.17	181.39	28.23	43.95	57.54	35.30	47.31	66.90
36-37	53	60.02	91.91	141.90	29.11	43.20	64.25	33.89	51.80	72.72
38-39	29	65.57	104.53	162.30	31.51	45.01	60.72	24.20	55.22	72.37
40-41	14	40.25	84.08	140.08	34.60	39.53	63.55	30.36	65.66	92.49

Pulsatility index										
16–19	63	0.79	1.09	1.64	1.12	1.38	1.92	1.34	1.66	2.21
20–23	63	0.60	0.90	1.38	1.01	1.25	1.47	1.40	1.71	2.33
24–27	63	0.58	0.85	1.17	0.88	1.08	1.34	1.47	2.01	3.04
28–31	63	0.51	0.72	1.11	0.79	1.03	1.27	1.52	2.12	2.97
32–35	63	0.50	0.72	1.47	0.66	0.91	1.18	1.52	2.12	2.91
36-37	53	0.43	0.67	1.30	0.56	0.84	1.16	0.87	1.75	2.51
38-39	29	0.46	0.67	1.09	0.63	0.79	0.98	1.22	1.72	2.29
40-41	14	0.62	0.78	1.55	0.63	0.78	1.10	1.36	1.68	2.78

GA, gestational age; P, percentile.

**Table 4:** Intra- and interobserver variability of the Doppler velocimetric measurements of PI and SV of some fetal arteries evaluated with their ICC in a cohort of low risk pregnant women.

Artery	Intraobserver		Interobserver	
	*N*	ICC (95% CI)	*N*	ICC (95% CI)
	PI			
UtA	268	0.757 (0.701–0.804)	129	0.826 (0.761–0.874)
MCA	272	0.617 (0.538–0.685)	129	0.391 (0.234–0.527)
UA	271	0.795 (0.746–0.835)	129	0.692 (0.590–0.772)

	SV			
UtA	268	0.812 (0.767–0.849)	129	0.248 (0.078–0.404)
MCA	272	0.891 (0.864–0.913)	129	0.926 (0.896–0.947)
UA	271	0.696 (0.629–0.752)	129	0.638 (0.552–0.730)

PI: pulsatility index; UtA: uterine artery; MCA: middle cerebral artery; UA: umbilical artery; ICC: Intraclass Correlation Coefficient; CI: confidence interval; SV: systolic velocity.
